# Co-Design Process of a Virtual Community of Practice for the Empowerment of People with Ischemic Heart Disease

**DOI:** 10.5334/ijic.5514

**Published:** 2020-11-09

**Authors:** Ana Toledo-Chávarri, Vanesa Ramos-García, Débora Koatz, Alezandra Torres-Castaño, Lilisbeth Perestelo-Pérez, Ana Belén Ramírez-Puerta, María-Eugenia Tello-Bernabé, Juan-Manuel García-García, Javier García-García, Valeria Pacheco-Huergo, Carola Orrego, Ana Isabel González-González, e-mpodera group

**Affiliations:** 1 Fundación Canaria Instituto de Investigación Sanitaria de Canarias (FIISC), Tenerife, ES; 2 Red de Investigación en Servicios de Salud en Enfermedades Crónicas (REDISSEC), ES; 3 Avedis Donabedian Research Institute (FAD), Barcelona, ES; 4 Universitat Autónoma de Barcelona, Barcelona, ES; 5 Servicio de Evaluación y Planificación del Servicio Canario de la Salud, Tenerife, ES; 6 Unidad de Apoyo Técnico, Gerencia Asistencial de Atención Primaria, Servicio Madrileño de Salud, Madrid, ES; 7 Gerencia Asistencial de Atención Primaria, Servicio Madrileño de Salud, Madrid, ES; 8 Unidad de Calidad y Seguridad del Paciente. Hospital Universitario Nuestra Señora de Candelaria, Tenerife, Canarias, ES; 9 Centro de Atención Primaria Turó, Instituto Catalán de la Salud, Barcelona, ES; 10 Institute of General Practice, Goethe University, Frankfurt, DE

**Keywords:** empowerment, virtual Communities of Practices, Ischemic heart disease, patient centred care, consumer involvement, co-design

## Abstract

**Introduction:**

Virtual Communities of Practices (vCoP) offer patients the possibility to interact and share tools and knowledge necessary for their empowerment. This paper describes the co-design process of a vCoP for the empowerment of people with ischemic heart disease (IHD).

**Methods:**

We used a modified experience-based design approach to co-design the vCoP in collaboration with people with IHD and health professionals consisting of two phases: exploratory and development phase. Data collection techniques included listening labs, workshops, and online participation.

**Results:**

Twenty-five people with IHD and ten health professionals participated. Experiences and needs for empowerment in IHD were identified in the exploratory phase allowing for the development of a Patient Journey Map. In the development phase, people with IHD prioritized needs to be addressed by the vCoP content framework in addition to content proposals.

**Discussion:**

The Patient Journey Map helped to easily visualize the empowerment needs of people with IHD and it might be transferable for the development of other people-centred interventions. The co-design process also allowed the development of training materials adapted to the priorities of people with IHD.

**Conclusion:**

A people-centred co-design process of a vCoP may facilitate the empowerment of people with IHD.

## Introduction

Ischemic heart disease (IHD) is the main cause of premature death among the population in Europe [1]. In Spain, approximately 30,000 people suffer a myocardial infarction every year [2], and among those who survive, around 20% go through a secondary cardiovascular event in the first year [3]. Regular exercise, a healthy diet, smoking cessation, and the use of cardiovascular preventive drugs are effective preventive actions that reduce mortality and re-infarction rates [4]. However, some people with IHD struggle when trying to reduce their cardiovascular risk factors [5]. Secondary prevention of IHD for reducing the impact of the disease after its onset is complex and challenging, thereby warranting the need to gain deeper knowledge on how to facilitate long lasting lifestyle changes.

Patient empowerment has been recognized as a key factor in improving health outcomes, increasing communication between patients and professionals, achieving better adherence to treatment, and ensuring an efficient use of primary health care resources [6]. The Health 2020 policy framework put forth by the World Health Organization (WHO) proposes strategies and action plans focused on investment in health through the empowerment of citizens [7]. In fact, a recent analysis on empowerment stated that *“it is a process that enables patients to exert more influence over their individual health by increasing their capacities to gain more control over issues they themselves define as important*” [8]. In people with IHD, autonomy and self-efficacy are considered relevant factors when planning their cardiac rehabilitation plan [9]. It has been shown that when people with IHD are autonomous and self-effective in managing their disease, they act to improve their lifestyle by adopting more preventive behaviours, such as making appropriate food choices and getting regular physical exercise [10].

Empowerment is also one of the principles of integrated care [11]. Coordination of care and services may improve by engaging people to actively participate in their care process and in the coproduction of integrated care interventions [12].

A virtual community of practice (vCoP) can be defined as a group of people with a common interest or problem who interact and share knowledge, learning together using an online platform that is developed on, and maintained using the Internet. vCoPs offer the possibility to identify and share resources and apply the tools and knowledge necessary for empowerment [13]. Through collaborative learning the experiences and knowledge of members support the community, strengthen relationships, and help raise ideas and innovation capacity in the area of interest [14]. The construction of knowledge is a social and interactive process. In this sense, learning communities are composed of three essential aspects: i) the existence of a space, physical or online, to share and build knowledge and capacities; ii) the mutual support among its members characterized by collaboration, interaction, and a feeling of belonging that facilitates the sharing of knowledge and experiences; and iii) the definition of learning as a process [15].

Co-design of interventions or health services can seek to effectively improve care [16]. A systematic review and meta-analysis of co-design research experiences [17] showed that co-designed interventions can improve health related outcomes both at individual and community levels, including physical health, healthy behaviours, self-efficacy, health service access and receipt, as well as strengthen community relations. The co-design elements that had the greatest impact on the effectiveness included, accommodating the interventions to the needs and priorities of their participants, and incorporating skills [17].

This paper describes the co-design of a vCoP for the empowerment of people with IHD, in collaboration with health professionals from primary and specialized care and a team of researchers. This work was conducted as part of the *e*-mpodera^2^ project, a randomized controlled trial that aims to evaluate the effectiveness and cost-effectiveness of the co-designed vCoP, as described herein, to improve patient activation and other measures related with patient empowerment in people with IHD (Trial registration: ClinicalTrials.gov, (NCT02757781), Registered on 25 April 2016).

## Research Methods

This study has been funded by the Carlos III Health Institute (Instituto de Salud Carlos III) through the project “PI18/01404, PI18/01397, PI18/01333”, and co-funded by the European Regional Development Fund (ERDF) “A way of shaping Europe”. It was approved by Clinical Research Ethics Committees of Gregorio Marañón University Hospital in Madrid (Acta 08/2019), Nuestra Señora de Candelaria University Hospital in Santa Cruz de Tenerife (CHUNSC_2019_05) and IDIAP Jordi Gol in Barcelona (19/053-P).

The present manuscript follows the reporting short checklist for PPI in research [18] (Additional file 1).

Additional file 1. https://doi.org/10.5334/ijic.5514.s1

### Design

**Figure 1:**
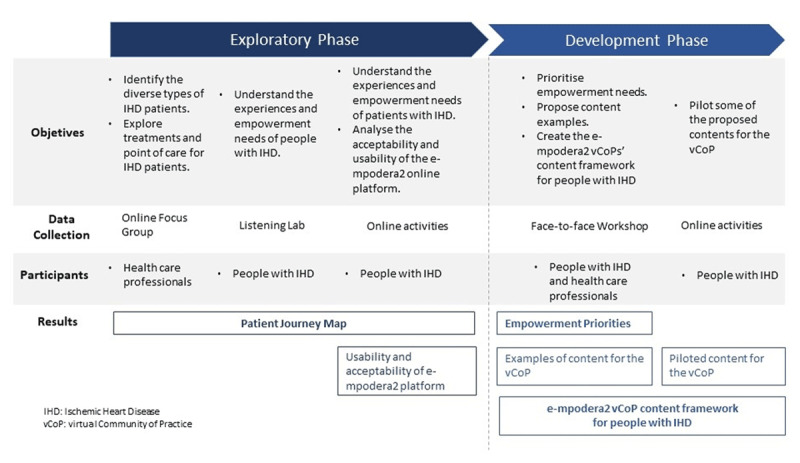
Co-design process.

We used a modified experience-based design approach [19] to co-design the *e*-mpodera^2^ vCoP intervention. The co-design process consisted of two sequential phases: i) exploratory phase, and ii) development phase. Specific objectives, data collection techniques, participants, and results for each phase are shown in Figure 1.

### Specific Objectives

The co-design process objective was to develop a structure and content examples for the randomized control trial vCoP. The specific objectives for each phase were:

1. The exploratory phase aimed: i) to identify different diagnosis and treatment paths for IHD; ii) to explore short, medium and long-term treatments and points of contact for people with IHD represented through a Patient Journey Map; iii) to understand the experiences and empowerment needs of people with IHD; and iv) to assess the acceptability and usability of the *e*-mpodera^2^ online platform.
2. The development phase sought: i) to prioritize addressable empowerment needs; ii) to propose examples of contents for the vCoP; iii) to create the *e*-mpodera^2^ vCoPs’ content framework for people with IHD; and iv) to pilot some of the proposed contents; and v) to continue the assessment of the acceptability and usability of the *e*-mpodera^2^ online platform.

### Setting

The study took place in the Spanish National Health System in three different regions in Spain: Canary Islands, Catalonia and Madrid.

### Participants

#### Inclusion criteria (people with IHD)

Age ≥ 18 years; diagnosis of IHD in the electronic medical record; be willing to be a member of the group and attend meetings for 6 months (at least one of the two face-to-face meetings and participate in the online co-design group); be willing to use Facebook, WhatsApp, Telegram or email within the framework of the co-design process; have signed the informed consent and commit to participate in the study (Additional file 2).

Additional file 2. https://doi.org/10.5334/ijic.5514.s2

#### Exclusion criteria (people with IHD)

Low probability of cooperation in the study, situation of displaced or being a temporary resident, institutionalized, with a terminal illness, or a physical or mental disability that limit their ability to comply with study requirements.

#### Inclusion criteria (health professionals)

General practitioners, practice nurses or cardiologists who were members of the research team or had expertise in cardiovascular diseases and/or patient empowerment. They ought to participate as part of the focus group or the development workshop.

#### Exclusion criteria (health professionals)

Not willing to participate in the study.

### Sample and recruitment

A purposive sample of people with IHD sought to include maximum variation regarding age, gender and time from onset. Recruitment of people with IHD in each region was conducted through IHD patient organizations and general practitioners, practice nurses, and cardiologists who showed interest in the study and who had participated in the previous research project *e*-mpodera^1^ [20,21], which developed a vCoP for primary care health professionals (general practitioners and practice nurses) to engage in patient empowerment.

A convenience sample of health professionals was recruited from *e*-mpodera^1^ participants and members of the research team who were not engaged in the design or facilitation of the co-design process. They were invited to participate in a focus group. Health professionals were not actively recruited for the development phase workshop, but participation was open for those wanting to contribute in the development of the vCoP.

Participation was voluntary. Participants were free to withdraw from the study at any time without affecting present or future medical treatment.

### Data collection

Facilitation for both phases was designed by a researcher specialized in PPI methods and conducted by a team of seven researchers with diverse experience in participation and qualitative research.

#### Exploratory phase

This phase comprised an online focus group with health professionals, three face-to-face listening labs with people with IHD in each of the included Spanish Regions (Canary Islands, Catalonia, Madrid), and online participation through the *e*-mpodera^2^ platform. Face-to-face labs were carried out in a paediatric hospital facility in the Canary Islands, in a research centre in Cataluña and in a primary care management facility in Madrid.

The online focus group with health professionals was an open discussion lead by a moderator and with an observer taking notes of mentioned topics. Listening labs were workshops based on deep listening qualitative techniques [22] where participants collaboratively drafted a Patient Journey Map built with cardboards.

We developed two semi-structured guides, one for the online focus group with health professionals (Table 1), and the other one for the listening labs with people with IHD (Table 2). Listening labs were followed by online participation in the *e*-mpodera^2^ platform where the randomized controlled trial vCoP will be held, a gamified web 2.0 platform, with interactive learning content [20,21]. The research team proposed two activities a week from 3^rd^ May to 7^th^ June 2019 and a moderator motivated people to comment, engage and participate. The description of the online participation activities can be found in Table 3.

**Table 1:** Focus Group guide for the exploratory phase with health professionals.


**Presentation (5 min)**
Name of the professional, specialty and association with empowerment.
**Define the stages of the Patient Journey Map (5 min)**
We predefined 4 stages of IHD to take into account in the Patient Journey Map: pre-diagnosis, diagnosis, treatment and follow-up. What would the key moments within these stages be that define patient care (e.g. pre-diagnosis: tests before first symptoms)?
**For each stage (5 minutes per stage)**
What is happening right now?
Where and how do patients access care?
What should patients do at this time (basic clinical aspects)?
**Empowerment needs**
The European EMPATHiE project defines the empowered patient as one who *“has control over the management of their disease/s on a daily basis, acts to improve their quality of life and has the necessary knowledge, skills, attitudes and self-perceptions to adjust their behaviour – and work in a partnership with others when necessary – to achieve optimal well-being.”*
The empowerment interventions are aimed at equipping patients (and their informal caregivers, when appropriate) with the ability to participate in decisions related to their disease to the degree they want, to become “co-managers” of their disease together with health professionals, develop self-confidence, self-esteem and skills to face the physical, emotional and social impact of the disease in their daily life.
**For each stage (less than 10 minutes per stage)**
What do you think are the main barriers to self-care and care in this stage?
What do they need to empower themselves? What information do they need?


**Table 2:** Listening labs guide for exploratory phase with people with IHD.


1. Presentation of the *e*-mpodera^2^ project and the dynamics of the workshop	
2. Questions for each of the stages: Diagnosis, Long-term follow-up and Post diagnosis	
What happened?/Clinical experience	Tell us your name and tell us briefly how your heart problem started (Diagnostic stage)
Treatment/recommendations	Who has performed rehabilitation? What was the experience with or without rehabilitation? (Post-diagnostic stage)
Contact points (who?)	What was the intervention and the recommendations?
What happens outside the doctor? Work environment – family	Who? Where? When?
Emotions	How was your life?
Empowerment Needs	What has changed?
Barriers to empowerment	What did you feel?
	What would you have liked to know?
	What would you have liked to be helped with?
	What problems did you face when taking care of yourself?
3. Present the Platform for online participation and the following phase of co-design	


IHD = Ischemic Heart Disease.

**Table 3:** Examples of online participation activities during the exploratory phase.


**Challenge 1:** Starting here (from the beginning)- Onboarding the *e*-mpodera^2^ platform	How to use the platform (tutorial) and participants’ introduction
**Challenge 2:** To upload an image of your recovery from IHD	We propose you to share photos that symbolize your experience in the recovery process of heart disease. Find or take a picture that represents some moment of the process and comment it with the following questions
**Challenge 3:** Healthy habits	In your experience, what have been the most difficult times to maintain your diet, exercise and/or not smoke? Give an example of the moment/s in which you have skipped the recommendations of healthy habits and comment on those of your partner. How have you overcome similar temptations?
**Challenge 4:** Reviewing the Patient Journey of IHD	The Patient Journey Map is a scheme that aims to reflect a path followed by people with ischemic heart disease. It is a summary of what we talked about in the first face-to-face session. We would like you to review this draft version. What do you think? Does it reflect well the general experience of having ischemic heart disease? Do you miss something?
**Challenge 5:** Communication skills	During your IHD process, do you remember any time when you had communication problems about the disease with someone in your family, partner, friends and/or the nearest circle? Share some experience and make a suggestion to that of another participant


IHD = ischemic heart disease.

#### Development phase

We conducted three face-to-face development workshops with people with IHD, one in each of the Spanish Regions, followed by online participation in the *e*-mpodera^2^ platform. We used a semi-structured guide for the workshops (Table 4). In Madrid, two health professionals participated in the content development; health professionals did not participate in the other two regions. Online participation consisted on the piloting of two co-designed contents each week between 10^th^ June and 12^th^ July 2019.

**Table 4:** Face-to-face Workshop Guide for development phase.


1.	Summary of what was done and presentation of the workshop dynamics
2.	Presentation of types of training resources of the virtual Community of Practice
3.	Prioritization of empowerment needs
4.	Proposals for training resources
5.	Evaluation of the acceptability and usability of the *e*-mpodera^2^ platform


### Analyses

The Patient Journey Map was progressively co-developed with people with IHD and health professionals. A unified draft was created with information from the focus group and the listening labs of the Exploratory phase. The research group reviewed participants input and proposed this unified version that was then reviewed by all participants. Points of contact, what happened, and treatment sections of the Patient Journey Map summarized both people with IHD and health professionals’ views; the feelings and empowerment needs were based on the input gathered from people with IHD. Only people with IHD participated in the prioritization of their needs.

To describe the experiences of people with IHD, we reviewed and summarized the listening labs notes.

We developed the *e*-mpodera^2^ vCoP’s content framework for people with IHD by adapting the prioritized empowerment needs and the materials proposed by the participants based on the EMPATHiE Consortium dimensions for patient empowerment [23]. The EMPATHiE Consortium dimensions have 3 pillars: i) the health literacy pillar considers the skills needed to find, understand, appraise, and apply information related to health and care services; ii) the self-management pillar aims to promote healthy lifestyle behaviours; and iii) the shared decision-making pillar which is defined as a decision-making process jointly shared by patients and their health care providers. To evaluate the acceptability and usability of the *e*-mpodera^2^ platform, we analysed the participants insights provided through open ended questions in development workshop after the participants had used platform for the online activities of the exploratory phase.

## Results

We conducted the co-design process between March and July 2019. Twenty-five people with IHD from the three Spanish Regions (eight, six and eleven from the Canary Islands, Catalonia, and Madrid, respectively), and ten health professionals (two, two, and six from the Canary Islands, Catalonia and Madrid, respectively) participated in the co-design process of the vCoP (Tables 5 and 6). Seven additional people with IHD participated in one activity but were lost to follow-up, so they were not considered full participants. The sample included people with IHD who showed strong knowledge and self-management skills (especially those who belonged to patient organizations), as well as people with self-reported low health literacy with respect to IHD. On average, participants with IHD were first diagnosed with IHD 5 years prior to the study.

**Table 5:** Characteristics of participants – people with IHD.

	Canary Islands	Catalonia	Madrid	Total

**Gender n**				
Female	1	1	3	5
Male	7	5	8	20
**Age (years) n**				
30–50	0	1	2	3
50–65	8	3	7	18
65+	0	2	2	4
**Age Mean (SD)**				57.4 (9.5)
**Time since diagnosis (years)**				
Less than 1	1	0	1	2
1–5	5	1	9	15
5+	2	5	1	8
**Time since diagnosis (years) Mean (SD)**				5.3 (4.2)

**Table 6:** Characteristics of participants – health professionals.

Region	Specialty	Sex	Participation

Canary Islands	Cardiologist	Male	Online
Canary Islands	General Practitioner	Male	Online
Catalonia	Cardiologist	Female	Online
Catalonia	General Practitioner	Female	Online
Madrid	General Practitioner	Male	Online
Madrid	General Practitioner	Male	Online
Madrid	Cardiologist	Female	Online
Madrid	General Practitioner	Male	Online
Madrid	Nurse Practitioner	Male	Workshop
Madrid	Nurse Practitioner	Female	Workshop

The co-design process involved two phases: i) the exploratory phase, where the experiences and needs for empowerment in IHD built the trajectory of care for the condition and ii) the development phase, where people with IHD prioritized the needs to be addressed by the vCoP, proposed training content for empowerment of people with IHD, facilitating the development of the *e*-mpodera^2^ vCoP’s content framework.

### Exploratory phase

**Figure 2:**
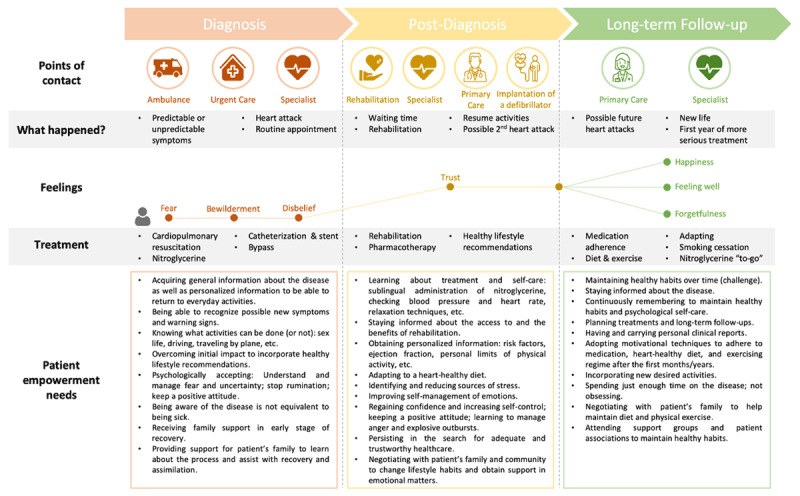
Patient Journey Map.

Points of contact, what happened, treatments, feelings, and empowerment needs for the three stages of the trajectory of care of people with IHD (diagnosis, post-diagnosis and long-term care) were visualised on the co-designed Patient Journey Map (Figure 2). The experiences of people with IHD and their empowerment needs were diverse depending on their gender, socio-economic and family situations, and on the impact the IHD had on their health, social and work life.

In the remainder of this section we describe the diversity of experience from people with IHD and identify empowerment needs on which the Patient Journey Map is based.

### Experiences of people with IHD

#### Diagnosis stage

The vast majority of people that participated were diagnosed with IHD after having suffered a myocardial infarction, while two were diagnosed during routine controls or follow-up of comorbid conditions. Not recognizing the symptoms of infarction was common. As the most widely known symptom of infarction was chest pain, people with IHD who did not experience that symptom tended to not recognize that they were having an infarct. Some thought they were suffering from anxiety or stomach bloating.

#### Post-diagnosis stage

Those participants who had a second infarction tended to immediately recognize the symptoms, as they did not vary from their first infarction. Infarction was followed by fear, bewilderment and disbelief that it happened to them.

People with IHD described the post-diagnosis stage as the months after the diagnosis where there is still some uncertainty about the adaptation to lifestyle changes and the learning of skills to best manage the cardiovascular disease. It was also a period in which participants stayed alert in regards with the disease and were motivated to change. Not all participants had access to rehabilitation services in this stage. This access depended on whether rehabilitation was available locally or the service had waiting lists. Participants, even those who had not received rehabilitation, considered it an essential milestone in the empowerment process.

#### Long-term follow-up stage

In the long-term follow-up stage, participants reported a decrease in alertness and motivation for change. This decrease usually occurred between one and two years after the infarction. After this one- or two-year period, which varied for each participant, it was more difficult to maintain heart-healthy habits.

### Empowerment needs

Empowerment needs varied in the diagnosis, post-diagnosis, and long-term stages as can be seen in the Patient Journey Map (Figure 2). We found needs can be classified in three categories: information and knowledge needs, needs related to psychological care, needs related to social support in care.

The main barriers to empowerment as described by people with IHD were consistent through all stages of the patient’s trajectory and were related to precarious socioeconomic or work conditions, lack of family support/accompaniment, low digital health literacy, or lack of access to adequate professional care or treatment (for example, due to coordination issues between physicians). Job and financial vulnerability made it difficult to access care, for example rehabilitation, which requires a large number of hours of attendance may require to take time off work. Gender inequality meant greater barriers to self-care and social support for women with IHD, as many of them had assigned caretaking roles in their families (e.g. with other dependents as child or elders).

#### Diagnosis stage

The identified needs included basic and general information of the IHD, possible symptoms and activities that can be carried out after a heart attack. Participants stated that the empowerment process required them to overcome the initial impact of receiving the diagnosis/of the infarction in order to incorporate healthy living recommendations, understand and manage the fear and uncertainty associated with the infarction, and assume the disease with a positive attitude. In relation to social support, the fundamental care network identified by the participants was their family who accompanied them throughout the trajectory of care. Caring was not always easy, and participants considered that family members and other carers needed support to recognize and to be able to help during the recovery process, including its early stages.

#### Post-Diagnosis stage

In the Post-Diagnosis stage, the information and knowledge needs shifted to personalized knowledge about the individual impact of the disease, knowledge about healthy activities adapted to each person’s circumstances, and about the lifestyle changes necessary for leading a heart-healthy life (regular exercise, healthy diet, and no smoking).

Regarding psychological needs, participants with IHD explained that it was necessary to become aware of the sources of stress and learn how to reduce them, as well as to improve their self-management of emotions. Lifestyle changes required social support and implied negotiations with family and other carers.

#### Long-term follow-up

In the long-term follow-up stage, participants, both people with IHD and health professionals, suggested that there was a need to use motivational techniques in order to encourage people with IHD to adhere to the recommendations regarding medication, diet, smoking cessation, and physical exercise after the first months or years. The maintenance of healthy habits also required negotiations with family and other carers.

For some participants, it was also empowering to build clinical support networks, that is, networks consisting of one or more health professionals to rely on in the long-term follow-up. For other participants, patient organizations were an important network of social support and contributed to the maintenance of long-term heart-healthy habits.

### Development phase

The development phase assessed the acceptability and usability of the *e*-mpodera^2^ platform from the perspective of people with IHD. Then, this phase focused on the prioritization of the empowerment needs and the formulation of both the *e*-mpodera^2^ content framework for people with IHD and contents for the vCoP.

#### Acceptability and usability of the e-mpodera^2^ platform

Most of the people with IHD found the *e*-mpodera^2^ platform acceptable and usable, with the exception of some older participants with self-reported low digital literacy. Participants suggested some changes to improve the platform, including adding a Spanish spell checker and emoticons in the comment section, improving tools for embedding videos, and a friendlier interface for voting and commenting. All these changes were implemented in order to improve usability.

#### Empowerment needs prioritization

The priority of empowerment needs varied by region, based on the main barriers experienced by the people with IHD in each context. In the Canary Islands, psychological needs were identified as the most relevant, while in Catalonia and Madrid the needs from all three categories were seen as equally relevant. We describe below the empowered needs that were prioritized in at least two groups.

In the diagnosis stage, people with IHD considered that it was a priority to begin to accept the diagnosis, including being able to recognize and overcome fear, uncertainty, and/or denial. They also pointed out that it is necessary to receive basic information about the disease (risk factors, causes, etc.), information on how to make the transition from the hospital to daily life, and information on the existence and benefits of rehabilitation. Finally, they identified family support as essential to help recovery and help take care of themselves.

In the post-diagnosis stage, the prioritized aspects were to improve the self-management of emotions to face the situation, regain confidence, and increase self-control. In addition, people with IHD pointed out the importance of receiving information on individualised risk factors and personal health information associated with the infarct (e.g. ejection fraction, number of vessels with lesions, residual ischemia, etc.), as well as recommendations on nutrition and recipes to maintain a heart-healthy diet. The main barriers perceived after diagnosis included work-related responsibilities, taking care of dependents, and other socioeconomic difficulties.

In the long-term follow-up stage, the main challenge was to maintain lifestyle changes over time and to connect with support groups and patient organizations with common objectives.

Prioritized empowerment needs were used by participants to elaborate example of contents for the CoP in the development workshop. The final description of empowerment needs included in the Patient Journey Map was revised and accepted by all participants in the online participation process.

#### The e-mpodera^2^ vCoP’s content framework for people with IHD

The *e*-mpodera^2^ vCoP’s content framework for people with IHD added, to the three aforementioned pillars, a fourth one based on contributions from the listening labs related to skills for the improvement of social and family support in care needs and practical issues.

The *e*-mpodera^2^ vCoP’s content framework, including examples of the content developed for the vCoP during the face-to-face workshops, is presented in Table 7. Some of these resources were transformed into online activities and were piloted on the platform. Supported by a moderator, two resources (interactive activity or challenge) were proposed online each week. Additionally, participants could open discussions at will and add content on their own. This was the case in four occasions, in which participants added ideas and commented on news related to IHD. These contents were added to the final material proposed for the vCoP.

**Table 7:** *e*-mpodera^2^ VCoP content framework for people with IHD with content examples.

Empowerment dimensions	Learning objectives	Examples of contents: co-created interactive activities and challenges

**Health literacy**		
Disease awareness	To improve awareness of ischemic disease, prevention and healthy lifestyle	Do you know how your heart works? What is ischemic heart disease? What does it consist of? Types. Risk factors. Glossary with key medical terms and their acronyms.
Communication skills	To improve communication skills	How to improve communication skills with the health professional and with your family?
Emotional management	To improve skills regarding emotional management	Tips for mental health How can I express my emotions to my family and ask for their support? Identify fear: make a list of the most fearful problems and seek support to overcome those fears
Knowledge about medicines management	To improve awareness about medicines management, as well as knowing the food-medicine interactions; drug interactions among others	What to know about medicines interaction? Mechanisms to avoid forgetting to take medication
Knowledge about healthy habits and lifestyle (food, exercise…) – habits change	To know resources for habit change	How to start changing habits? Food labelling: Fats, sugar, salt. Leave behind myths: Healthy diet: there is still confusion about the use of salt and coffee.
**Self-management**		
Healthy food	To get resources and recipes for healthy food	How to read labels. Recipes from famous people with ischemic disease Healthy recipes competency
Exercise and physical activity – sport	To get advice and routines for applying physical exercise	What if I want to dedicate myself to my usual physical exercise, despite my illness?
Stop smoking and drinking alcohol	To share resources for smoke and alcohol cessation	12 Tips to quit smoking
Self-tracking (weight, emotions, sleeping, walking…)	To know how to self-track measures	Basic information on physical self-control resources. Essential questions: Control and monitoring of heart rate. Resources for self-control: pulsations, blood pressure monitor, pedometer.
Symptoms recognition	To improve awareness about symptoms recognition	To prepare cardiologist consultation
Emotional care	To improve emotional care	Psychological care: Tools to promote relaxation: videos/recordings of Jacobson’s relaxation, mindfulness, yoga.
FAQs (Asking professionals)	To offer a consulting resource for asking professionals doubts, and other uncomfortable questions	Answers for uncomfortable or frequent questions
**Shared Decision Making**		
Living will	To decide how to manage willing last decisions in different scenarios	How to treat awkward issues with carers and family How to speak about living will with family and carers
Preferences about treatment and specific daily decisions	To improve awareness about treatment possibilities, recovery available and other daily decisions.	What to know about recovery options
Recovery decisions preferences	To improve awareness about recovery options	How to prepare cardiologist’s visit: Preparing a list of questions
**Social and family support and practical issues**		
Return to normal life (social and family activities, and relationships, back at work)	To manage skills to get progressively to normal life, getting support from the social environment, family and work. To improve family awareness to support relatives with ischemic disease	Family communication: facilitate two-way communication to improve how I communicate with my family and how they communicate with me.
Economic and practical issues	To manage difficulties at work and/or economic problems after disease	How to manage problems at work and practical issues Share resources, patients’ organizations, expert advice and other tools

IHD = ischemic heart disease.

## Discussion

The co-design process allowed people with IHD to share their experiences and empowerment needs, as visualized in the Patient Journey Map. We adapted the mapping technique to elicit empowerment needs from the perspective of people with IHD. Patient Journey Maps are increasingly used in person-centred health service design, particularly in association with quality improvement processes but the technique is scarcely used in health research [24,25]. We have not found any previous study using a Patient Journey Map to co-design a vCoP, with the exception of Coy et al. (2019) [26] who used a similar design. More specifically, their work used experience-based co-design to visually represent the experience of children and their families, and suggested areas for improvement that helped the design of an educational tool.

Our co-design process also enabled the development of multiple contents adapted to people with IHD needs and priorities that will help to develop the *e*-mpodera^2^ vCoPs. The *WHO Framework on Integrated People-Centred Health Services* encourages co-production of health services that can meet people’s needs in the long term and are coordinated both within and beyond the health care system [29]. Co-design processes may serve to develop health services that promote both person-centred and integrated care [29]. We believe this Patient Journey Map and the its inclusion in co-design processes may be transferable and can be adapted to design person-centred and integrated health services for IHD such as rehabilitation, lay educational materials, and other self-management interventions.

### Strengths and limitations

Patient Journey Maps help to easily visualise, share, and co-produce knowledge [24]. The IHD Patient Journey Map allowed us to show the changing empowerment needs in the diagnosis, post-diagnosis, and long-term care stages. This processual perspective helped to elicit empowerment needs beyond the urgency of a myocardial infarction.

The co-design process established a partnership between people with IHD, health care professionals, and researchers that helped develop the *e*-mpodera^2^ vCoPs’ content framework. Participants produced an enormous quantity of ideas for training resources and activities, and most of them will be used in the randomized controlled trial to test the effectiveness of the vCoP for improving patient’s empowerment. vCoPs are highly participative and dynamic, and the final contents will depend on the interest and needs of its actual participants.

Lack of economic and human resources, barriers for participants’ engagement and retention, and other problems can hinder co-design processes [16]. Ideally, the co-design processes should include a maximum variation sample to incorporate a diverse range of experiences. Perspectives of women, elderly, minorities, migrants, and socially excluded groups may add specificities that will not be collected if they are not included in a participatory process [17]. Nevertheless, maximum variation of the sample was not achieved in our co-creation process. A limited number of women with IHD (n = 8/25) participated. Their experiences and needs are specific [25,27] and were underrepresented in the co-design process. Furthermore, all participants, except one, had experienced a myocardial infarction so other experiences were not included. We were not able to recruit minorities, migrants, and socially excluded groups due to lack of time and resources. Older and less digitally literate participants found the *e*-mpodera^2^ platform difficult to use and some participants did not conclude the process. As noted by Greenhalgh et al. (2017) [28] older, sicker, and less digitally literate people are less likely to benefit from teleservices such as a vCoP. Engagement of people with IHD in the conceptualization, design and recruitment of this study might have helped to recruit more and more diverse participants.

Another limitation of this study was the lack of training for the research group in participation facilitation, which was easily solved by capacity building within the group.

## Conclusion

The co-design process conducted in this study was new and exciting for the *e*-mpodera group. It resulted in a strong collaboration between the research team, people with IHD, and health professionals. The co-design process resulted in a Patient Journey Map that helps to easily visualise the empowerment needs of people with IHD in diagnosis, post-diagnosis, and long-term care stages. Additionally, the Patient Journey Map could potentially be adapted in the development of person-centred interventions. Co-design also allowed us to develop training materials adapted to the needs and priorities of people with IHD. The process generated many more content ideas than expected, ultimately generating a rich and extensive framework for the *e*-mpodera^2^ vCoP.

## Additional Files

The additional files for this article can be found as follows:

10.5334/ijic.5514.s1 Additional file 1. GRIPP2 short form checklist.

10.5334/ijic.5514.s2 Additional file 2. Informed consent.

## Author Information

The *e*-mpodera research group is formed by the authors and Sofía Garrido-Elustondo, (Gerencia Asistencial Atención Primaria, Servicio Madrileño de Salud, Red de Investigación en Servicios de Salud en Enfermedades Crónicas (REDISSEC), Juan Carlos Obaya-Rebollar (Centro de Salud Chopera, Gerencia Asistencial de Atención Primaria), Santiago Díaz-Sánchez (Centro de Salud Pintores, Gerencia Asistencial de Atención Primaria), Luis Morales-Cobos (Centro de Salud Las Américas, Gerencia Asistencial de Atención Primaria), Ana Rodríguez-Almodovar (UGC Cardiología, Hospital Universitario Reina Sofía), Juana Mateos-Rodilla (Escuela Madrileña de Salud, Dirección General de Humanización y Atención al Paciente), Marta Ruiz López (CS Vicente Muzas, Gerencia Asistencial de Atención Primaria, Servicio Madrileño de Salud), Yolanda Canellas (CS Monovar, Gerencia Asistencial de Atención Primaria, Servicio Madrileño de Salud), José Ignacio Vicente-Díaz (CS Monovar, Gerencia Asistencial de Atención Primaria, Servicio Madrileño de Salud), José Carlos del Castillo (Área de prevención y medicina cardiovascular. Hospital San Juan de Dios), Nuria Vallejo-Camazón (Hospital Germans Trias i Pujol) Marta Ballester (Avedis Donabedian Research Institute (FAD), Barcelona, Universitat Autónoma de Barcelona, Red de Investigación en Servicios de Salud en Enfermedades Crónicas).
