# Supplementary material for: Co-Design Process of a Virtual Community of Practice for the Empowerment of People with Ischemic Heart Disease
Source: Int J Integr Care. 2020 Nov 9;20(4):9. doi: 10.5334/ijic.5514 (PMC7664302; doi:10.5334/ijic.5514)
Supplement: Additional file 1. — GRIPP2 short form checklist. [file ijic-20-4-5514-s1.pdf]

Additional file 1. GRIPP2 short form checklist

**Section and topic**	**Item**	**Section in which reported**
1: Aim	Report the aim of PPI in the study	Introduction and methodology
2: Methods	Provide a clear description of the methods used for PPI in the study	Methodology
3: Study results	Outcomes – report the results of PPI in the study, including both positive and negative outcomes	Results
4: Discussions and conclusions	Outcomes: Comment on the extent to which PPI influenced the study overall. Describe positive and negative effects	Discussion and conclusions
5: Reflections / critical perspective	Comment critically on the study, reflecting on the things that went well and those that did not, so others can learn from this experience	Discussion

PPI = Patients and Public Involvement
