# Supplementary material for: Co-Design Process of a Virtual Community of Practice for the Empowerment of People with Ischemic Heart Disease
Source: Int J Integr Care. 2020 Nov 9;20(4):9. doi: 10.5334/ijic.5514 (PMC7664302; doi:10.5334/ijic.5514)
Supplement: Additional file 2. — Informed consent. [file ijic-20-4-5514-s2.pdf]

Additional file 2. Informed consent

HOJA DE INFORMACIÓN AL PACIENTE

FASE CO-DISEÑO

INTRODUCCIÓN

Estimado/a Sr/a:

Le comunicamos que se está desarrollando la primera fase de co-creación de la intervención para la puesta en marcha de un estudio posterior más amplio o ensayo clínico denominado "Efectividad y coste-efectividad de una intervención virtual (Comunidad de Práctica) para la mejora del empoderamiento de pacientes con cardiopatía isquémica en atención primaria: ensayo controlado aleatorizado por conglomerados" (Cataluña: PI18/01404/, Madrid: PI18/01397, Canarias: PI18/01333).

Este estudio ha sido aprobado por los Comités Éticos de los centros participantes de acuerdo a la legislación vigente, la Ley Orgánica 3/2018, de 5 de diciembre de Protección de Datos Personales y garantía de los derechos digitales, y a la aplicación del Reglamento (UE) 2016/679 del Parlamento europeo y del Consejo de 27 de abril de 2016 de Protección de Datos (RGPD) por el que se regula este tipo de estudios.

Nuestra intención es tan solo que usted reciba la información correcta y suficiente para que pueda evaluar y juzgar si quiere o no participar en este estudio. Para ello lea esta hoja informativa con atención y nosotros le aclararemos las dudas que le puedan surgir después de la explicación. Además, puede consultar con las personas que considere oportuno.

PARTICIPACIÓN VOLUNTARIA

Debe saber que su participación en este estudio es voluntaria y que puede decidir no participar o cambiar su decisión y retirar el consentimiento en cualquier momento, sin que por ello se altere la relación con su médico ni se produzca perjuicio alguno en su tratamiento.

¿QUIENES SON LOS INVESTIGADORES?

El equipo de investigación está formado por un equipo multidisciplinar (medicina, psicología, estadística y evaluación de servicios sanitarios, médicos de familia, enfermeras, cardiólogos) cuyos miembros pertenecen a las siguientes instituciones: Fundación Avedis Donabedian, Gerencia Asistencial de Atención Primaria (GAAP) del Servicio Madrileño de Salud y Servicio de Evaluación del Servicio Canario de la Salud (SESCS). Este proyecto ha surgido de una iniciativa colaborativa en el marco de la Red de Investigación en Servicios de Salud en Enfermedades Crónicas (REDISSEC).

DESCRIPCIÓN GENERAL DEL ESTUDIO

¿Por qué se hace este estudio?

Para ayudar a definir los componentes y contenidos específicos de una Comunidad de Práctica (CdP) virtual dirigida a pacientes con cardiopatía isquémica (CI), así como la estrategia a seguir en su evaluación. El objetivo del ensayo clínico posterior es evaluar la efectividad de una CdP virtual en la mejora del empoderamiento de pacientes con cardiopatía isquémica en atención primaria.

¿Quién puede participar?

Si usted es >18 años, tiene cardiopatía isquémica, dispone de internet en su hogar y/o Smartphone y está dispuesto a formar parte del grupo y a la asistencia a reuniones y participar a través de Facebook, Whatsapp, Telegram, y/o correo electrónico durante 6 meses, es usted es candidato a participar en este estudio.

Si usted desea participar, ¿en qué consiste su participación?

Los participantes (pacientes y profesionales) participarán en la co-creación de una plataforma virtual que será la intervención a evaluar en el ensayo clínico.

Se prevé reclutar a 6 profesionales sanitarios (3 por CC.AA.) y 15 pacientes (5 pacientes por CC.AA).

¿Cuánto tiempo requerirá la participación?

La participación en este estudio requerirá la asistencia a dos reuniones presenciales distribuidas en 6 meses a partir de abril de 2019 con una duración de 2 horas y media aproximadamente. Asimismo, se requerirá la asistencia a tres talleres *online* de una duración no superior a 1 hora.

En las sesiones presenciales, el consentimiento informado deberá ser firmado a mano en el momento de llegada a la sesión.

En los casos donde su participación es *online*, en la pantalla del ordenador y/o Smartphone aparecerá el consentimiento informado en el que deberá hacer “click” para dar su conformidad en la participación.

RIESGOS Y BENEFICIOS DE LA PARTICIPACIÓN EN ESTE ESTUDIO

No se prevé ningún tipo de riesgo físico ni psicológico que pueda ser consecuencia de la participación en este estudio.

No obstante, diversas investigaciones han mostrado cómo los pacientes que entienden su enfermedad y participan en la toma de decisiones sobre su salud con sus profesionales sanitarios se benefician del proceso, tomando decisiones más acordes con sus valores y preferencias.

El principal beneficio para los pacientes con CI contribuir a mejorar su conocimiento, habilidades y autoconfianza para la gestión de su propia salud y de la asistencia sanitaria.

CONFIDENCIALIDAD

El tratamiento, la comunicación y la cesión de los datos de carácter personal de todos los sujetos participantes se ajustará a lo dispuesto en la Ley Orgánica 3/2018, de 5 de diciembre de Protección de Datos Personales y garantía de los derechos digitales, y a la aplicación de del Reglamento (UE) 2016/679 del Parlamento europeo y del Consejo de 27 de abril de 2016 de Protección de Datos (RGPD), por lo que es importante que conozca la siguiente información:

- Además de los derechos que ya conoce (acceso, modificación, oposición y cancelación de datos) ahora también puede limitar el tratamiento de datos que sean incorrectos, solicitar una copia o que se trasladen a un tercero (portabilidad) los datos que usted ha facilitado para el estudio. Para ejercitar sus derechos, diríjase al investigador principal del estudio. Le recordamos que los datos no se pueden eliminar aunque deje de participar en el estudio para garantizar la validez de la investigación y cumplir con los deberes legales y los requisitos de autorización de medicamentos. Así mismo tiene derecho a dirigirse a la Agencia de Protección de Datos si no quedara satisfecho/

- Tanto el Centro como el Promotor y el Investigador son responsables respectivamente del tratamiento de sus datos y se comprometen a cumplir con la normativa de protección de datos en vigor. Los datos recogidos para el estudio estarán identificados mediante un código, de manera que no se incluya información que pueda identificarle, y sólo su médico del estudio/colaboradores podrá relacionar dichos datos con usted y con su historia clínica.
Por lo tanto, su identidad no será revelada a ninguna otra persona salvo a las autoridades sanitarias, cuando así lo requieran o en casos de urgencia médica. Los Comités de Ética de la Investigación, los representantes de la Autoridad Sanitaria en materia de inspección y el personal autorizado por el Promotor, únicamente podrán acceder para comprobar los datos personales, los procedimientos del estudio clínico y el cumplimiento de las normas de buena práctica clínica (siempre manteniendo la confidencialidad de la información).

El Investigador y el Promotor están obligados a conservar los datos recogidos para el estudio al menos hasta 25 años tras su finalización. Posteriormente, su información personal sólo se conservará por el centro para el cuidado de su salud y por el promotor para otros fines de investigación científica si usted hubiera otorgado su consentimiento para ello, y si así lo permite la ley y requisitos éticos aplicables.

INFORMACIÓN ADICIONAL

Tal y como exige la ley, para participar deberá firmar y fechar el documento de consentimiento informado.

COORDINADORA DEL PROYECTO (CATALUÑA):
Dra. Carola Orrego, Fundación Avedis Donabedian
Contacto: corrego@fadq.org

INVESTIGADORA PRINCIPAL (MADRID):
Ana Isabel González González, Centro de Salud Vicente Muzas, Gerencia Asistencial de Atención Primaria. Servicio Madrileño de Salud
Contacto: aisabel.gonzalezg@salud.madrid.org

INVESTIGADORA PRINCIPAL (CANARIAS):
Lilisbeth Perestelo Pérez, Servicio de Evaluación del Servicio Canario de la Salud
Contacto: lperperr@gobiernodecanarias.org

CONSENTIMIENTO INFORMADO PARA PACIENTES

Yo (nombre y apellidos)

....

He leído la hoja de información que se me ha entregado.
He podido hacer preguntas sobre el estudio.
He recibido suficiente información sobre el estudio.
He hablado con:

....

Comprendo que mi participación es voluntaria.
Comprendo que puedo retirarme del estudio:

1º Cuando quiera
2º Sin tener que dar explicaciones.
3º Sin que esto repercuta en mis cuidados médicos.

- Presto libremente mi conformidad para participar en el estudio y doy mi consentimiento para el acceso y utilización de mis datos en las condiciones detalladas en la hoja de información.

Nombre del participante: Nombre del investigador:

Fecha: Fecha:

Firma: Firma:
